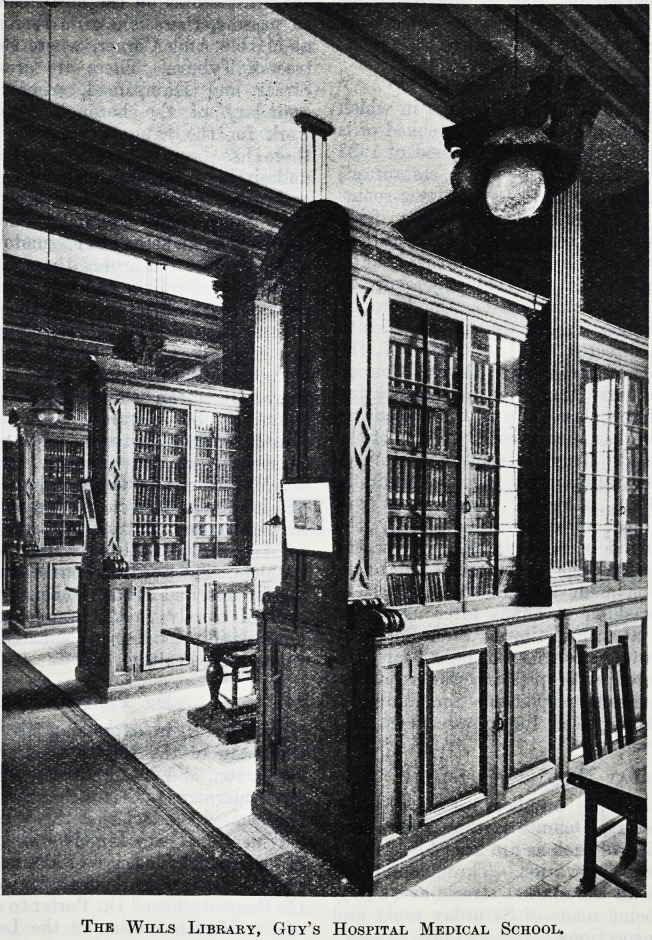# A Medical School Library

**Published:** 1924-09

**Authors:** 


### A MEDICAL SCHOOL LIBRARY.

One of the primary needs of the specialist is a specialist library. Knowledge grows with extraordinary rapidity, and every addition to it means more books, more magazines, more contributions to professional literature. The discovery of the connection of the mosquito with malaria, for instance, has produced a considerable literature ; the problems raised by the study of psychology have during the last few years produced shelves full of books. The medical student, no less than the medical specialist, has need of the latest and most authoritative works upon the many subjects which go to his equipment, and it is obvious that every great teaching hospital needs its own library, constantly replenished and kept up to date. Guy's Medical School has been exceptionally fortunate in this respect, since it possesses the splendidly fitted Wills Library, the gift of the late Sir Frederick Wills, Bart., M.P., which was opened in the summer of 1903. It is large enough to accommodate eighty-five students at the same time, and is arranged in twelve bays with a table and chairs in each. It contains some 10,000 volumes—books of reference, standard text-books and the best medical and surgical journals—and is under the care of a librarian. Students may make use of it from 9.30 a.m. to 5 p.m. each day. Situated on the ground floor of the Main School Building to the right of the entrance, it is adjoined by a room containing a collection of objects of historical interest relating to the hospital, its founder and its medical school, and by the Gordon Museum of pathological anatomy. Our illustration gives some idea of the character of the handsomely carved bookcases and the studious aspect of the room.

The Wills Library, Guy's Hospital Medical School.

## Figures and Tables

**Figure f1:**